# Supplementary material for: Disulfide disruption reverses mucus dysfunction in allergic airway disease
Source: Nat Commun. 2021 Jan 11;12:249. doi: 10.1038/s41467-020-20499-0 (PMC7801631; doi:10.1038/s41467-020-20499-0)
Supplement: Supplementary file 1 — Supplementary Information [file 41467_2020_20499_MOESM1_ESM.pdf]

Supplementary information

Disulfide disruption reverses mucus dysfunction in allergic airway disease

Leslie E. Morgan[1,*], Ana M. Jaramillo[1,*], Siddharth K. Shenoy[2,3], Dorota Raclawska[1], Nkechinyere A. Emezienna[1,4], Vanessa L. Richardson[1], Naoko Hara[1], Anna Q. Harder[1], James C. NeeDell[1], Corinne E. Hennessy[1], Hassan M. El-Batal[5], Chelsea M. Magin[1,5], Diane E. Grove Villalon[6], Gregg Duncan[2,7], Justin S. Hanes[2,3,8,9], Jung Soo Suk[2,3,9], David J. Thornton[10], Fernando Holguin[1], William J. Janssen[1,11,12], William R. Thelin[6], Christopher M. Evans[1,12]

[1] Department of Medicine, School of Medicine, University of Colorado, Aurora, CO, USA

[2] Center for Nanomedicine at the Wilmer Eye Institute, Johns Hopkins University School of Medicine, Baltimore, MD, USA

[3] Department of Ophthalmology, Johns Hopkins University School of Medicine, Baltimore, MD

[4] Department of Department of Obstetrics and Gynecology, Howard University College of Medicine, Washington, DC, USA

[5] Department of Bioengineering, College of Engineering, Design, and Computing, University of Colorado, Denver | Anschutz Medial Campus, Denver, CO, USA

[6] Parion Sciences, Inc., Durham, NC, USA

[7] Fischell Department of Bioengineering, School of Engineering University of Maryland, College Park, MD, USA

[8] Department of Pharmacology & Molecular Sciences, Johns Hopkins University School of Medicine, Baltimore, MD, USA

[9] Department of Chemical & Biomolecular Engineering, Johns Hopkins University, Baltimore, MD

[10] Wellcome Trust Centre for Cell-Matrix Research and the Lydia Becker Institute of Immunology and Inflammation, School of Biological Sciences, The University of Manchester, Manchester, UK

[11] Department of Medicine National Jewish Health, Denver, CO, USA

[12] Department of Immunology and Microbiology, School of Medicine, University of Colorado, Aurora, CO, USA

[*] Both authors contributed equally to this work

Corresponding author:
Christopher M. Evans, PhD
Professor
Division of Pulmonary Sciences and Critical Care Medicine
Department of Medicine
University of Colorado Denver School of Medicine
12700 E 19th Avenue, Mailstop 8611, RC 2
Room P15-3121
Aurora, CO 80045
(303) 724-6573 [tele]
Christopher.Evans@cuanschutz.edu

Supplementary Table 1. Lavage leukocyte totals in AEC analysis.

	**Measure**	**Vehicle, n = 6 mice**	**TCEP, 500 mM, n = 8 mice**	**p**
**Total cells**	mean (*sem*)	15.6 (*3.8*)	5.8 (*0.8*)	0.03^{t}
	median (*quartiles*)	13.7 (*7.7-23.5*)	4.8 (*4.2-6.9*)	0.006^{U}
**Macrophages**	mean (*sem*)	8.0 (*1.8*)	4.2 (*0.8*)	0.05^{t}
	median (*quartiles*)	7.5 (*4.9-10.7*)	3.5 (*2.6-6.1*)	0.04^{U}
**Eosinophils**	mean (*sem*)	5.0 (*0.2*)	1.0 (*0.3*)	0.04^{t}
	median (*quartiles*)	3.1 (*2.1-8.2*)	0.6 (*0.2-1.9*)	0.003^{U}
**Lymphocytes**	mean (*sem*)	1.4 (*0.5*)	0.5 (*0.08*)	0.07^{t}
	median (*quartiles*)	0.8 (*0.6-2.2*)	0.5 (*0.3-0.7*)	0.02^{U}
**Neutrophils**	mean (*sem*)	1.2 (*0.8*)	0.2 (*0.08*)	0.1^{t}
	median (*quartiles*)	0.4 (*0.2-1.9*)	0.1 (*0.05-0.3*)	0.05^{U}

^{t} Welch's t test, one-tailed
^{U} Mann-Whitney U-test, one-tailed

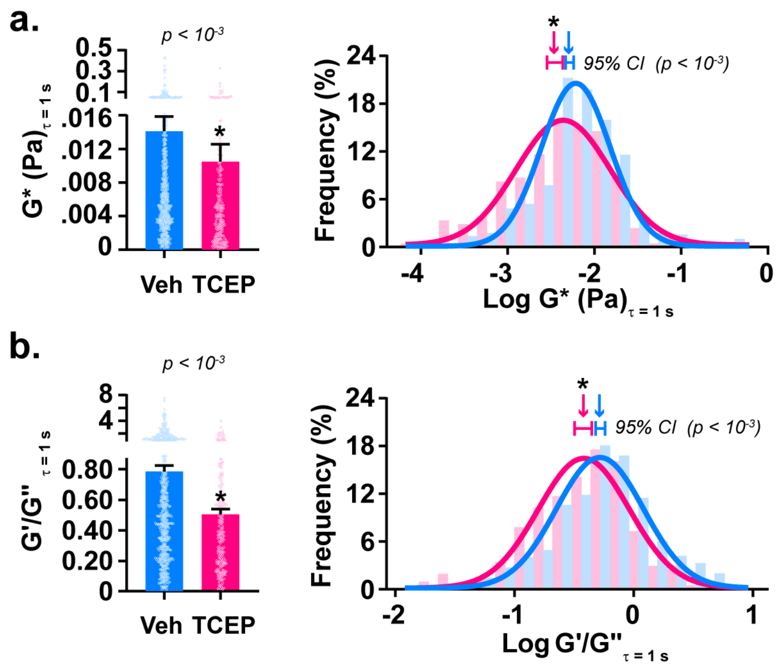

Supplementary Fig. 1. TCEP reduces mucus viscoelasticity. Multiple particle tracking was used to estimate mucus rheology. Carboxylated 2 μm diameter polystyrene micro-particles were applied to mucus and incubated in the presence of TCEP (10 mM, magenta, n=205 technical replicates) or saline vehicle (Veh, cyan, n=549 technical replicates) for 30 min at 37° C. MSD values were mathematically converted to complex (G*), elastic (G'), and viscous (G'') moduli. Compared to Veh controls, G* (**a**) and G'/G" ratio (**b**) decreased significantly after TCEP. Bars are means ± sem, shown with individual points (open circles). Histograms show log distributions of particles with Gaussian fits, with horizontal bars indicating 95% confidence intervals and inverted arrows indicating median values. Significance was determined by two-tailed Mann-Whitney U tests with p-values shown and '*' denoting significance between TCEP and Veh treated samples. Source data are provided as a Source Data file.

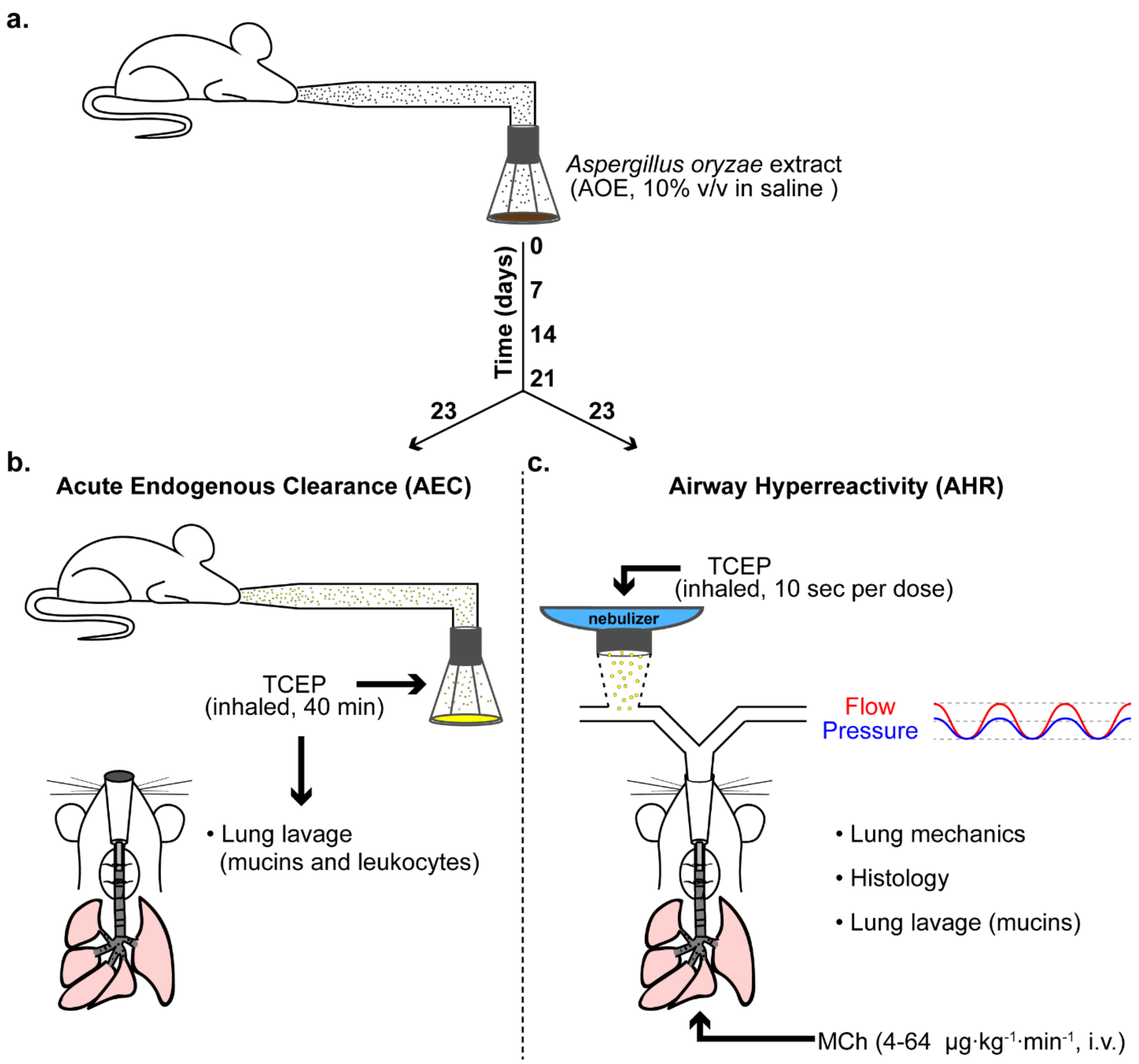

Supplementary Fig. 2. Mouse models used for *in vivo* studies. a. Wild type (WT) BALB/cJ mice were challenged by nose-only aerosol with *Aspergillus oryzae* extract (AOE) diluted 1:10 (vol/vol) in saline. A total volume of 5 ml was delivered over a 40 min period on days 0, 7, 14, and 21. Control mice received nose-only aerosols containing saline only. Mice were used for testing acute endogenous clearance (AEC) or airway hyperreactivity (AHR) two days after their last AOE challenge (day 23). **b.** For AEC, TCEP (500 mM) or saline vehicle was delivered in a 5 ml aerosol for 40 min. Mice were immediately used in lung lavage studies, and inflammatory cell numbers were enumerated. **c.** For AHR, lung mechanics and AHR to methacholine (MCh) were assessed in the presence of TCEP (100 mM) or saline vehicle, followed by determination of mucus plugging and mucin disruption.

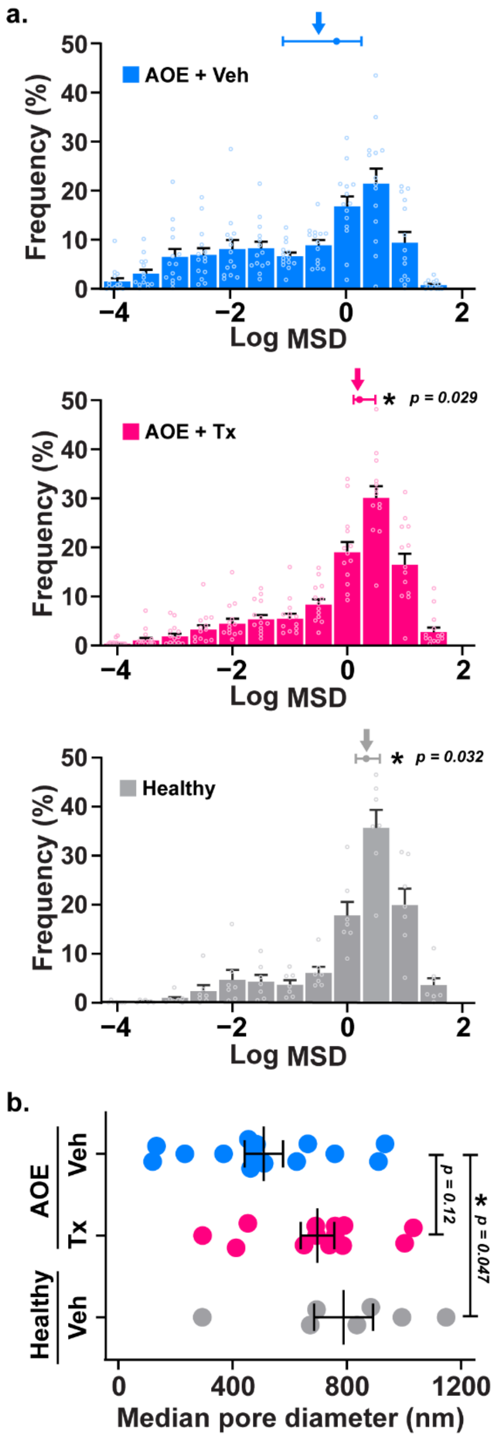

Supplementary Fig. 3. Microstructural properties affected by TCEP in allergic mouse mucus *in vivo*. **a.** Mean-squared displacement (MSD) measurements from diffusion of 100 nm muco-inert nanoparticles (MIPs) in tracheal mucus were used to calculate median pore sizes. Bars are medians ± interquartile ranges. Inverted arrows indicate the average for each set. **b.** Compared to healthy mice (grey, n = 7 mice), mucus shifted towards tighter pore sizes with AOE exposure (cyan, n = 14 mice). TCEP treatment (Tx), increased mucus pore sizes in AOE challenged mice (magenta n = 13 mice) to be comparable to the healthy state. Values are means ± sem. P-values were determined by one-way ANOVA with Tukey's test for multiple comparisons. Source data are provided as a Source Data file.

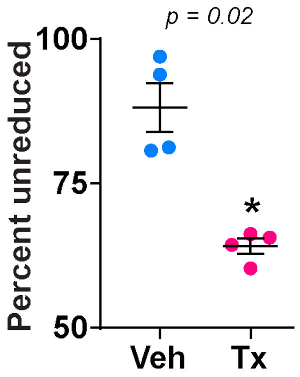

Supplementary Fig. 4. Relative quantification of mucin polymers from mice in AEC studies. Intensities of high molecular weight (polymerized) and low molecular weight (depolymerized) mucins were evaluated using Image Studio software (Li-Cor). Demarcations of polymerized and depolymerized signals are indicated in **Fig. 2d**. Data are presented as the fraction of polymerized versus total (polymerized and depolymerized) signals per lane. TCEP treatment (Tx, magenta, n=4 mice) increased mucin depolymerization compared to Veh controls (cyan, n=4 mice). Data are means ± sem. Significance was determined by one-tailed Mann-Whitney U test with p-value shown and '*' denoting significance ($p < 0.05$) from Veh. Source data are provided as a Source Data file.

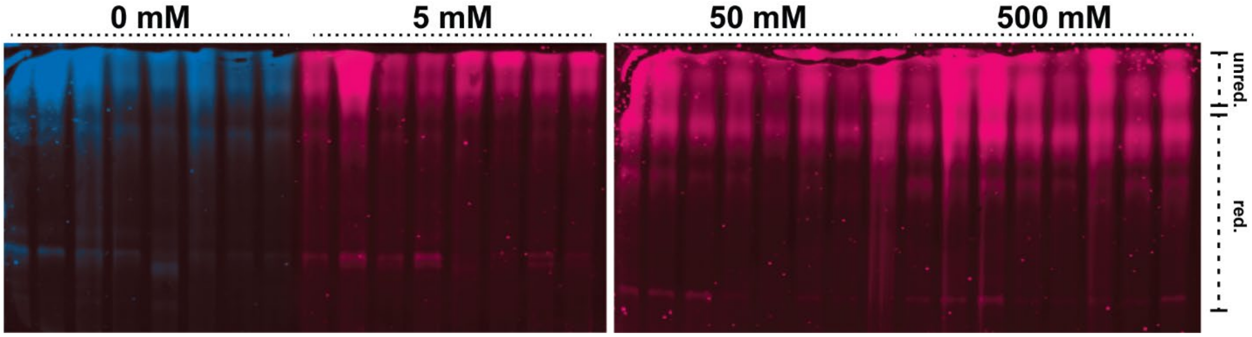

Supplementary Fig. 5. Concentration dependent effects of TCEP on mucin depolymerization. Lung lavage fluid from AOE challenged mice was obtained after treatment by nose-only aerosol with TCEP (0 mM, cyan; 5 mM, 50 mM, and 500 mM, magenta, n = 8 mice per dosage group). Blot was probed with UEA-1 (α1,2-fucose). Image shows 8 samples per group. Data in blot image were used for quantification in **Fig. 2e**. Source data are provided as a Source Data file.

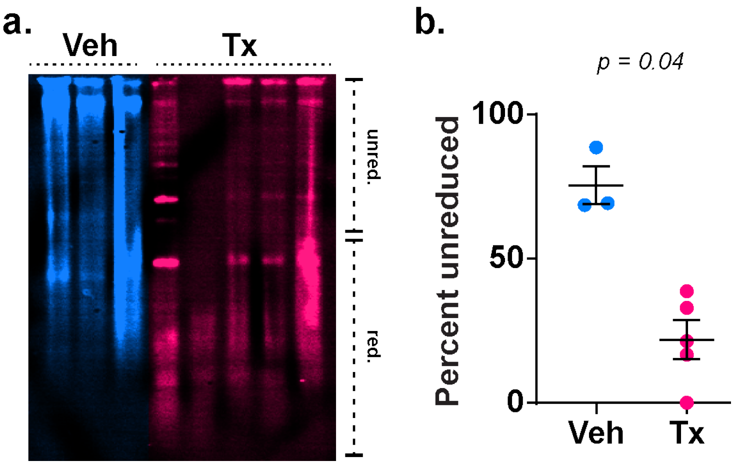

Supplementary Fig. 6. Immunoblot analysis of mucolytic effects in lung lavage fluid from allergic mice after AHR analysis. Lung lavage fluid from AOE challenged mice was obtained at the end of AHR studies. Equal volumes of lavage fluid (25 µl) were loaded per well in 1% SDS agarose gels. Samples were separated by electrophoresis, vacuum transferred to PVDF membranes, blotted with rabbit-anti-Muc5b, and detected with anti-rabbit IgG conjugated with Alexa 680. **a**. Relative to saline vehicle controls (Veh, cyan, n=3 biological replicates), Muc5b migration was enhanced by TCEP treatment (Tx, magenta, n=5 biological replicates). **b**. Intensities of high molecular weight (polymerized) and low molecular weight (depolymerized) mucins were evaluated using Image Studio software (Li-Cor). Demarcations of polymerized and depolymerized signals are indicated in **a**. Data are presented as the fraction of polymerized versus total (polymerized and depolymerized) signals. Data in **b** are means ± sem. Significance was determined by one-tailed Mann-Whitney U test with p-value shown and '*' denoting significance ($p < 0.05$) from Veh. Source data are provided as a Source Data file.

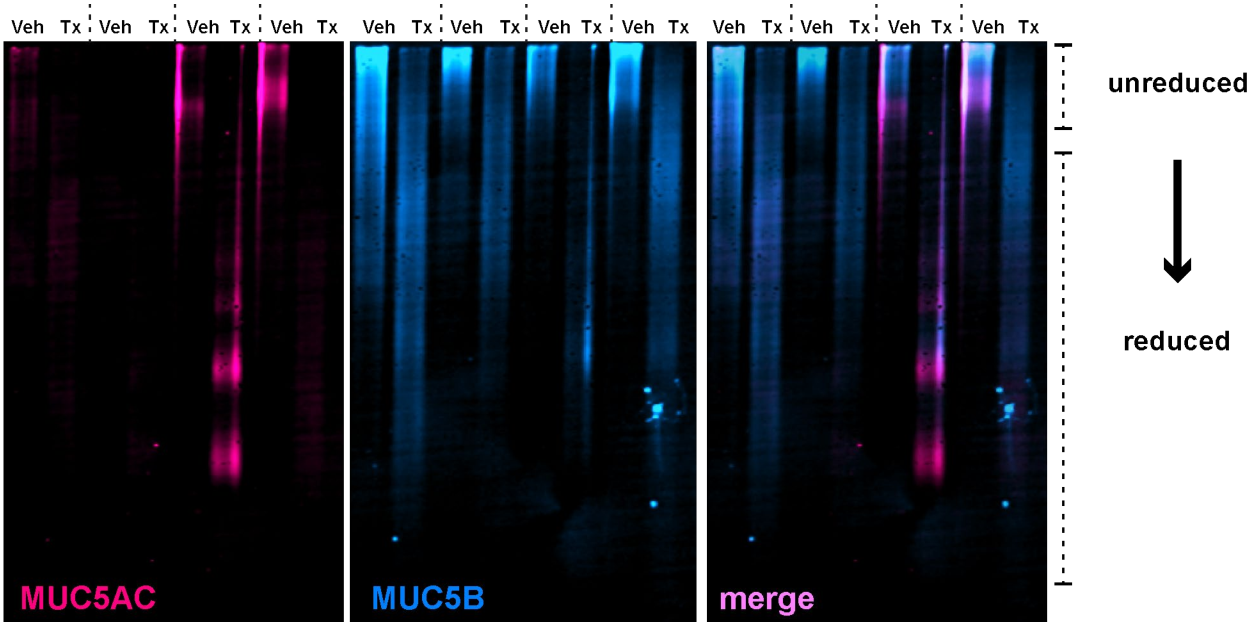

Supplementary Fig. 7. TCEP depolymerizes MUC5AC and MUC5B in bronchoalveolar lavage fluid from human asthma patients. Rabbit-anti-MUC5AC (magenta) and goat-anti-MUC5B (cyan) antibodies were applied (1:5,000 dilution) and developed using anti-rabbit-IgG conjugated with Alexa-680 and anti-goat-IgG conjugated with Alexa 800. Samples from n=4 patients with asthma were treated with TCEP (10 mM final concentration) at neutral pH for 10 min at 37°C. Note that full reduction causes epitope loss for both anti-mucin antibodies, resulting in diminished signal intensities. Source data are provided as a Source Data file.

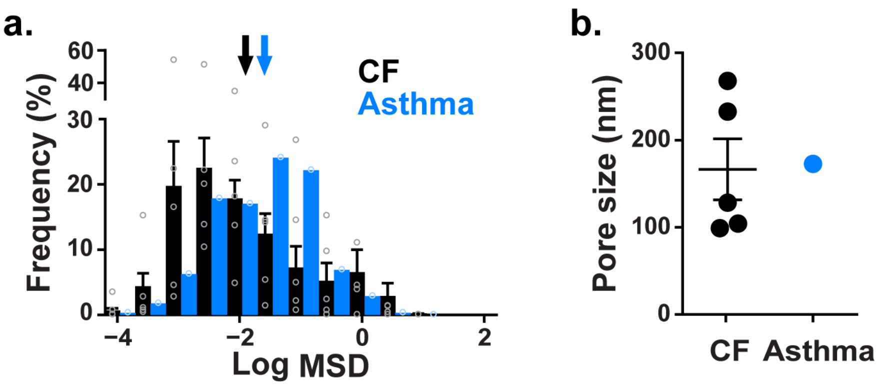

Supplementary Fig. 8. Histogram and median pore sizes derived based on diffusion of 100 nm muco-inert nanoparticles in spontaneously expectorated sputum from cystic fibrosis patients and in mucus aspirated from bronchial explants from a fatal asthma patient. Cystic fibrosis **(**CF) sputum (n = 5) and asthma (n =1). **a.** Distribution of the log median MSD's of individual muco-inert nanoparticles (MIPs). Data represent at least 500 particles tracked per sample. Inverted arrows indicate the average for each set. The tracking resolution is Log (MSD) of -3 or MSD values of 10^{-3} μm^2. Bars indicate means ± sem of log median MSD's, with circles identifying individual median log MSD's for each replicate **b.** Median pore sizes in asthma mucus appear comparable to range in CF mucus. Data are means ± sem with dots showing individual sample medians in **b**. Mucus solids concentrations were 7.1 ± 0.5% (CF) and 7.7% (asthma). Source data are provided as a Source Data file.
