# Supplementary material for: Disulfide disruption reverses mucus dysfunction in allergic airway disease
Source: Nat Commun. 2021 Jan 11;12:249. doi: 10.1038/s41467-020-20499-0 (PMC7801631; doi:10.1038/s41467-020-20499-0)
Supplement: Supplementary file 3 — Reporting Summary [file 41467_2020_20499_MOESM3_ESM.pdf]

nature research

Corresponding author(s): Christopher Evans

Last updated by author(s): 6 Nov 2020

Reporting Summary

Nature Research wishes to improve the reproducibility of the work that we publish. This form provides structure for consistency and transparency in reporting. For further information on Nature Research policies, see Authors & Referees and the Editorial Policy Checklist .

Statistics

For all statistical analyses, confirm that the following items are present in the figure legend, table legend, main text, or Methods section.

n/a	Confirmed	
☐	☒	The exact sample size (*n*) for each experimental group/condition, given as a discrete number and unit of measurement
☐	☒	A statement on whether measurements were taken from distinct samples or whether the same sample was measured repeatedly
☐	☒	The statistical test(s) used AND whether they are one- or two-sided<br>*Only common tests should be described solely by name; describe more complex techniques in the Methods section.*
☒	☐	A description of all covariates tested
☐	☒	A description of any assumptions or corrections, such as tests of normality and adjustment for multiple comparisons
☐	☒	A full description of the statistical parameters including central tendency (e.g. means) or other basic estimates (e.g. regression coefficient) AND variation (e.g. standard deviation) or associated estimates of uncertainty (e.g. confidence intervals)
☐	☒	For null hypothesis testing, the test statistic (e.g. *F*, *t*, *r*) with confidence intervals, effect sizes, degrees of freedom and *P* value noted<br>*Give P values as exact values whenever suitable.*
☒	☐	For Bayesian analysis, information on the choice of priors and Markov chain Monte Carlo settings
☒	☐	For hierarchical and complex designs, identification of the appropriate level for tests and full reporting of outcomes
☒	☐	Estimates of effect sizes (e.g. Cohen's *d*, Pearson's *r*), indicating how they were calculated

Our web collection on statistics for biologists contains articles on many of the points above.

Software and code

Policy information about availability of computer code

Data collection	Code used for measuring mucus biophysical properties is available as an open source. Microscopy performed on an Olympus microscope was performed using Olympus cellSens software (v 2.3), lung mechanics was performed on a flexiVent using flexiWare (v 7.6)
Data analysis	Data analyses were performed using commercially available GraphPad software (v 8.4.3). Immunoblot images were acquired and anlyzed using Li-COR Image Studio software (v5.x CLx).

For manuscripts utilizing custom algorithms or software that are central to the research but not yet described in published literature, software must be made available to editors/reviewers. We strongly encourage code deposition in a community repository (e.g. GitHub). See the Nature Research guidelines for submitting code & software for further information.

Data

Policy information about availability of data

All manuscripts must include a data availability statement. This statement should provide the following information, where applicable:

- Accession codes, unique identifiers, or web links for publicly available datasets
- A list of figures that have associated raw data
- A description of any restrictions on data availability

Source Data are included with this manuscript

Field-specific reporting

Please select the one below that is the best fit for your research. If you are not sure, read the appropriate sections before making your selection.

☒ Life sciences ☐ Behavioural & social sciences ☐ Ecological, evolutionary & environmental sciences

For a reference copy of the document with all sections, see nature.com/documents/nr-reporting-summary-flat.pdf

Life sciences study design

All studies must disclose on these points even when the disclosure is negative.

Sample size	In C57BL/6J mice, MCh dose response curves had slopes of 0.57±0.54 and 1.87±0.98 (mean ± s.d.) in saline and AOE groups, respectively. PMID: 25687754. Power analysis suggests 8 mice per group (www.dssresearch.com). We estimated 7-10 mice per group to observe differences at α = 0.05 and ≥80% power.
Data exclusions	No data were excluded.
Replication	Technical and biological replicates were studied on different days (>100 days). All attempts at replication were successful.
Randomization	Mice, samples, and datasets were randomized for evaluations. For histological quantification randomized design-based stereology was performed.
Blinding	Experimenters were blinded to genotypes and to treatment groups for all allergen vs control treatment groups in all mouse AHR and mucus clearance studies on days of experiments. MPT values were analyzed with blinding as to the treatment groups. Histological mage analysis was done blinded in all cases.

Reporting for specific materials, systems and methods

We require information from authors about some types of materials, experimental systems and methods used in many studies. Here, indicate whether each material, system or method listed is relevant to your study. If you are not sure if a list item applies to your research, read the appropriate section before selecting a response.

Materials & experimental systems

n/a	Involved in the study
☐	☒ Antibodies
☒	☐ Eukaryotic cell lines
☒	☐ Palaeontology
☐	☒ Animals and other organisms
☐	☒ Human research participants
☒	☐ Clinical data

Methods

n/a	Involved in the study
☒	☐ ChIP-seq
☒	☐ Flow cytometry
☒	☐ MRI-based neuroimaging

Antibodies

Antibodies used	For immuno-detection, human and mouse mucins were detected using rabbit-anti-human MUC5AC (MAN5AC, made by Dr. Thornton, not commercially available), mouse-anti-MUC5AC (clone 45M1, Catalog #MA5-12178, ThermoFisher), rabbit-anti-human MUC5B (H300, Catalog #sc-20119Santa Cruz), and rabbit-anti-mouse Muc5b (made by Dr. Evans, not commercially available. Secondary antibodies used were a goat anti-mouse IRDye 680RD (Li-Cor, Cat #926-68070) or goat anti-rabbit IRDye 800CW (Li-Cor, Cat #926-32211) antibodies were observed in the manuscript.
Validation	Knockout mouse samples were used for mouse-anti-MUC5AC (PMID: 23187315) and rabbit-anti-mouse Muc5b antibodies (PMID: 24317696). For human samples, molecular masses on agarose gels were indicative of very high molecular weight mucin polymers (>1 MDa). Anti human antibodies have also been validated by western blot and dot blot ELISA using MUC5AC and MUC5B knockout A549 cells. Data not shown. According to the vendor, 45M1 cross-reacts with Cat, Human, Mammal, Mouse, Non-human primate, Pig, Rabbit, Rat (>100 citations). H300 is no longer available commercially, but in practice we have found that it cross-reacts minimally with mouse, rat, and ferret tissues.

Animals and other organisms

Policy information about studies involving animals; ARRIVE guidelines recommended for reporting animal research

Laboratory animals	Male and female BALB/cJ wild type mice were purchased from the Jackson Labs (Bar Harbor, ME). Muc5ac /- mice were previously crossed onto a congenic BALB/cJ strain background. Animals were housed under specific pathogen-free conditions and used in allergic asthma studies beginning at ages 6-8 weeks.
Wild animals	The study did not involve wild animals.
Field-collected samples	The study did not involve samples collected in the field.
Ethics oversight	IRB and IACUC panels oversaw research at the University of Colorado, National Jewish Health, and Johns Hopkins.

Note that full information on the approval of the study protocol must also be provided in the manuscript.

Human research participants

Policy information about studies involving human research participants

Population characteristics	Bronchoalveolar lavage samples from 4 Patients with stable asthma were used (2 M, 2 F). The patients were enrolled for a separate IRB approved study and anonymized samples were stored in a Universtiy of Colorado biobank. Subjects had no history of infection, steroid use, or asthma-related hospitalization within 4 wks of study<br><br>Expectorated sputum was collected from 5 volunteers (3 M, 2 F) with CF as part of an unrelated study at Johns Hopkins. Discard material was used for the studies reported here. No patients were on CFTR modulator therapies, all CF patients had histories of positive bacterial cultures wihtin 1 year of study, 4 of 5 showed evidence of bacterial infection at time of study. CF mutations were: F508del/F508del, F508del/R1066C, F508del/W1282X, F508del/L88X, and F508del/exon 2 del.<br><br>All patients were adults, non-pregnant, non-smokers.

Recruitment	Adult volunteers were recruited based on disease characteristics. Sex and age were not used as selection criteria. Volunteers must have been able to participate in a study that required two visits for baseline and study days within two weeks. For fatal asthma donors, there was no additional selection criteria.
Ethics oversight	IRB panels at the University of Colorado, National Jewish Health, and Johns Hopkins.

Note that full information on the approval of the study protocol must also be provided in the manuscript.
